# Preliminary Study on the Effectiveness of Short Group Cognitive Behavioral Therapy (GCBT) on Indonesian Older Adults

**DOI:** 10.1371/journal.pone.0057198

**Published:** 2013-02-21

**Authors:** Dharmayati Utoyo Lubis, Edo Sebastian Jaya, Retha Arjadi, Lathifah Hanum, Kresna Astri, Maha Decha Dwi Putri

**Affiliations:** Faculty of Psychology, University of Indonesia, Depok, Indonesia; Federal University of Rio de Janeiro, Brazil

## Abstract

This research aims to develop evidence based affordable psychological therapy for Indonesian older adults. An affordable psychological therapy is important as there is virtually no managed care or health insurance that covers psychological therapy in Indonesia. Multicomponent group cognitive behavior therapy (GCBGT) was chosen as a starting point due to its extensive evidence, short sessions, and success for a wide range of psychological problems. The group format was chosen to address both the economic and the cultural context of Indonesia. Then, the developed treatment is tested to common psychological problems in older adults' population (anxiety, chronic pain, depression, and insomnia). The treatment consists of 8 sessions with twice a week meetings for 2.5 hours. There are similarities and differences among the techniques used in the treatment for the different psychological problems. The final participants are 38 older adults that are divided into the treatment groups; 8 participants joined the anxiety treatment, 10 participants for the chronic pain treatment, 10 participants for depression treatment, and lastly, 10 participants joined the insomnia treatment. The research design is pre-test post-test with within group analysis. We used principal outcome measure that is specific for each treatment group, as well as additional outcome measures. Overall, the result shows statistical significance change with large effect size for the principal outcome measure. In addition, the result for the additional measures varies from slight improvement with small effect size to statistically significant improvement with large effect size. The result indicates that short multicomponent GCBT is effective in alleviating various common psychological problems in Indonesian older adults. Therefore, multicomponent GCBT may be a good starting point to develop an effective and affordable psychological therapy for Indonesian older adults. Lastly, this result adds to the accumulating body of evidence on the effectiveness of multicomponent GCBT outside western context.

## Introduction

Indonesia is a developing country located in South-East Asia. But, like other developed countries, Indonesia is experiencing a greying of the population as the baby-boomers generation of the 60 s are becoming older adults. There is a gradual increase of older adults' percentages in the population every year. The last census showed that older adults have reached 7.59% of the population (18.04 million people)[1]. This quite sudden population change captured the attention of Indonesian mental health professionals to address future mental health problems of the older adults'.

The sudden population change calls for the need of providing psychological therapy that is accessible to older adults. Currently, there is virtually no managed care or health insurance system that covers psychological therapy in Indonesia. The cost of psychological therapy in Indonesia is entirely burdened on the client. Even in a developed country like the United States, self-pay for psychotherapy is too expensive[2]. To make matters worse, many Indonesian older adults no longer have stable source of income and rely on monthly allowance from their children. This economical condition pushes the need for the presence of economical psychological therapy.

To address the issue of affordable psychological therapy, we develop a psychological therapy with short sessions. Multicomponent cognitive behavioral therapy (CBT) is the first that comes to mind as it is currently one of the most evidence based approach of psychological therapy. Multicomponent CBT is a psychological therapy that combines behavioral and cognitive techniques, as well as other evidence based technique (such as: relaxation). The goal of the therapy is for an adaptive change of the behavioral, affective, and cognitive aspect of the client[3]. Multicomponent CBT has been shown to be effective for numerous psychological problems[4]. It has also been shown to be effective with short sessions[5].

Another relevant issue in delivering psychological therapy in Indonesia is their distinctive culture. Indeed, addressing the cultural context of psychological therapy has been recognised as essential for an effective therapy[6], [7]. Therefore, this research will adopt elements of Indonesian culture in delivering the therapy. One of the most known element of Indonesian culture is called collective culture[8]. The Indonesian collective culture is often expressed in their habit for social gatherings[9], sometimes termed as *arisan* or *silaturahmi*. Many Indonesians engage in social gatherings every day with different people, while most have at least 2 scheduled social gatherings every week. International students of western background are surprised at how often Indonesians engage in social gathering, which is not possible to do in their home country[10]. They are surprised at the amount of time that Indonesians have, and that it is mostly spent on social gatherings. The group setting of this therapy will take advantage of Indonesian collective culture. This group setting hopes to emulate their social gathering habit and make the therapeutic setting to be more natural.

The efficacy of the treatment will be tested on the common psychological problems in the older adults' population. Psychological problems with high prevalence in the older adults' population are anxiety, chronic pain, depression, and insomnia[11]. Anxiety occurs in about 15%–20% of community samples of older adults[12]. While chronic pain occurs at up to 50% of community dwelling older adults [13]. On the other hand, depression prevalence is estimated to be at the rate of 16% of older adults in U.S. [14]. Finally, insomnia is estimated to be present at the rate of up to 50% of older adults in a study of 9,000 Americans[15].

This research aims to conduct a preliminary investigation on the effectiveness of GCBT approach on the common psychological problems of Indonesian older adults. This research is important because investigation on GCBT outside western context has been relatively low[6]. We conducted a literature search and found minimal result that investigated CBT on South East Asian context. We only found an article that investigated the effectiveness of GCBT on depression in Malaysia[16]. We did not find investigation on the efficacy of CBT for older adults in South East Asia. Being the first of its kind, this research hope to shed light on two main problems, which is to help provide evidence based short term psychological therapy for Indonesian older adults and to investigate the efficacy of GCBT in the context of Indonesian older adults.

## Methods

### Ethical statement

This research was approved by the Ethical Committee of Faculty of Psychology, University of Indonesia, Indonesia. Oral informed consent was given for the screening questionnaire. Then, an additional written informed consent was given for the treatment participants.

### Research design

The research is a quasi-experimental design of pre-test post-test with within group analysis [17]. There are four treatment groups with a focus on different psychological problems (anxiety, chronic pain, depression, and insomnia). There is a standard set of questionnaire that is administered to all treatment groups and further accompanied by a specific set of questionnaire that is unique to each treatment group. The participants are measured at baseline before treatment and one week after the treatment for post-test.

Before treatment, the participants are given informed consent to inform them about the nature of the research and that no personal data will be published. Furthermore, the participants received a small incentive in exchange for their travelling cost to the research site. As the participants are attracted in public settings, all of the participants are willing to participate and get improved.

### Participants

Participants of this research are older adults that are identified as having anxiety, chronic pain, depression, or insomnia problems. In addition, they are willing to go through eight session of group cognitive-behavioural therapy (GCBT). Furthermore, the participants are given informed consent that stated their rights, data privacy, and their volunteer act. Initially, there are 43 participants that filled the pre-test assessment, signed informed consent, and confirmed to join the treatment. But, there are only 38 participants (88.38%) who completed post-test assessment. There are three participants that decided to stop the treatment and two participants did not come for the post-test. The total participants' age range from 47 to 79 years old, with the mean of 63.42 years old (SD = 7.25). There were 27 female participants (71%) and 11 male participants (29%).

All 38 participants were separated into four treatment groups: anxiety, chronic pain, depression, and insomnia. Each participant was offered to join one of the therapy group based on their major psychological problem/complaint and/or their preferences to join one of the treatment groups. From the total number of participants, there were 8 participants (22%) for anxiety, 10 participants (26%) for chronic pain, 10 participants (26%) for depression, and 10 participants (26%) for insomnia treatment group. The five participants who did not finish treatment are dropped out from further analysis. There were two participants from chronic pain treatment group, two participants from depression treatment group, and one participant from insomnia treatment group that is dropped out.

### Procedure

**Figure 1:**
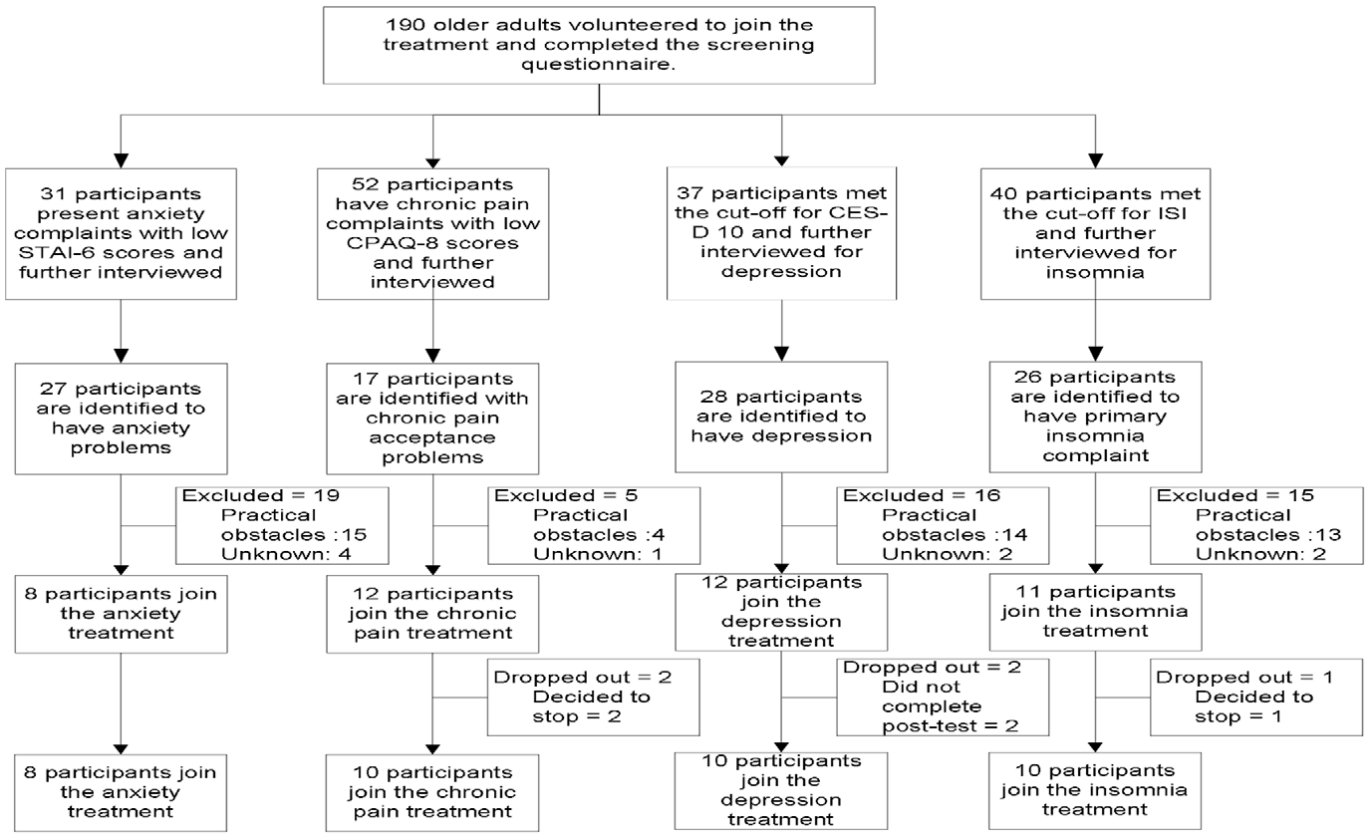
STAI-6: State-Trait Anxiety Inventory 6; CPAQ-8: Chronic Pain Acceptance Questionnaire 8; CES-D 10: Center for Epidemiologic Studies Depression Scale 10; ISI: Insomnia Severity Index.

We attract research participants by going to several gathering points of Indonesian elderly in Depok, West Java, Indonesia. Most of the gathering points that we visit are a location for group sport activity (*senam*). After the elderly group finished their physical exercises, we give an announcement about the research. Those who wanted to participate in the treatment are asked to fill in a set of questionnaires that included their contact detail. The questionnaires are then collected and those who met the cut-off for the questionnaire is contacted to be briefly interviewed and invited to participate in the therapy. In addition, the participants who met the cut-off for more than one therapy are given a choice to select the therapy that is most useful to them at the present. The participants' selection process is shown in Figure 1.

All of the treatments consist of 8-sessions that are done twice a week, with 3–4 days between sessions from session 1 to 7. The last session is the post-test session, with a full week break after the last session. Each session is held at 2.5 hours at regular time. The pre-test is conducted at the first session (session 1) and the post-test is done at the last session (session 8).

### Measures

There are several measures used to measure the treatment efficacy. For each treatment, there are principal outcome measures and secondary measures. However, there is a main set of measures that is always used. The main set measures are the State-Trait Anxiety Inventory 6 (STAI-6), Chronic Pain Acceptance Questionnaire 8 (CPAQ-8), Center for Epidemiological Studies Depression Scale (CES-D), Insomnia Severity Index (ISI), Perceived Stress Questionnaire (PSQ), and Subjective Well-Being scale (SWB). The additional measures include Penn State Worry Questionnaire (PSWQ) for anxiety treatment, Beck Depression Inventory (BDI) for depression treatment, and sleep diary indicators for insomnia treatment.

We used the short form version for all of the measures (except ISI, PSQ, and SWB) to improve brevity. There are no short form versions available for the PSQ, ISI, and SWB. But, the ISI only consist of 7 items and SWB only has 3 items. Brevity of the measures was of particular important, as fatigue is an important consideration on research with the elderly population.

Furthermore, all of the measures are adapted through back-translation process by a sworn translator to Bahasa Indonesia. Afterwards, two licensed clinical psychologists who specialize in gerontology scrutinize the measures further. Then, after further revision, the measures are tested to a non-psychology background elderly for readability. Finally, the adapted measures are printed in large fonts (Times New Roman 15 and 18).

#### Anxiety measures

State-Trait Anxiety Inventory (STAI), specifically STAI-6, is used as the principal measure for anxiety. STAI originally consists of 20 items[18] and is the most widely used subjective measures of anxiety in health research [19]. We used STAI-6, the short form, to improve brevity of the questionnaire. Furthermore, STAI-6 is highly correlated with the full version of STAI and has a good reliability[19]. The STAI-6 consists of 6 items, with 4-point Likert scale, from 1  =  almost never to 4  =  almost always[20].

Moreover, Penn State Worry Questionnaire (PSWQ) is also used as an additional anxiety measure. PSWQ is widely used to assess pathological worry in both clinical and non-clinical populations. It was developed to evaluate the disposition to worry and its frequency, the excess or intensity of worry, and the tendency for worry to be generalized. PSWQ is not restricted to one or a small number of situations. It consists of 16 items with 5-point Likert scale from 1  =  not at all typical of me to 5  =  very typical of me[21].

#### Chronic Pain Acceptance Questionnaire 8 (CPAQ-8)

The principal outcome measure used for the chronic pain treatment is the Chronic Pain Acceptance Questionnaire 8 (CPAQ-8). CPAQ originally has 20 items and is commonly used to measure acceptance of pain with 7-point Likert scale, from 0  =  never true to 6  =  always true[22]. There are two principal factors in CPAQ: activities engagement and pain willingness. Activities engagement measures how individual's pain restricts them to do their daily activities, and pain willingness measures individual's efforts to control their pain[23]. The short version of CPAQ has 4 items for each factor[24].

#### Depression measures

The main measure for depression is the Center for Epidemiologic Studies Depression Scale (CES-D). CES-D is a self-report 20 items questionnaire that measures depressive symptomatology in general population, which includes positive affect, depressed or negative affect, somatic symptoms, and interpersonal problems[25], [26].It has high internal consistency and adequate test-retest repeatability[25]. We used the short version of CES-D with 10 items (CES-D 10) for brevity. The cut-off score to indicate depression for the CES-D 10 is 10[27]-[29]. The CES-D items are filled according to the frequency of symptoms experienced. It has 4-point Likert scale, which includes 0  =  less then 1 day, 1  =  1–2 days, 2  =  3–4 days, to 3  =  5–7 days[25].

We also used the adapted Indonesian Beck Depression Inventory-I (BDI-I) to measure depression from behavioral, emotional, and somatic symptoms[26], [30]. BDI-I has been adapted to Indonesian language, content, and norms[31]. It has 21 items, which consists of several statements. The participants are to choose one of the statements that best describe their condition. The score for each item range from 0 to 3. The cut-off score for the Indonesian BDI-I is 6 to 14 for mild to moderate depression and 15 and above for severe depression[31].

#### Insomnia measures

The principal insomnia measure is the Insomnia Severity Index (ISI), and is further accompanied by several indicators from sleep diary. The ISI measures an overall indicator of insomnia, such as: severity of sleep problems, sleep satisfaction, daytime impairments, visibility of sleep problems, and distress due to sleep problems[32]. ISI used 5-point Likert scale from 0  =  None to 4  =  Very Severe. The score ranged from 0 to 28 and is classified into 4 categories: no insomnia (0–7), sub-threshold insomnia (8–14), moderate severity clinical insomnia (15–21), and severe clinical insomnia (22–28). ISI can be a good indicator of treatment effectiveness[33].

The ISI measure is accompanied by sleep diary to assess the insomnia treatment outcome. Sleep diary is used to obtain self-report measure of time before sleep onset, total wake time after sleep onset, number of times awake, total sleep time, sleep efficiency, feeling refreshed after sleep, and sleep quality. Sleep diary have good validity and reliability to gather information regarding the client's sleep pattern, even comparable to polysomnography[32]. Furthermore, sleep diary is often used to test the effectiveness of treatment[34].

The time before sleep onset variable is the estimated time needed to fall asleep after going to bed. The total wake time after sleep onset is the amount of total time spent in being awake (minutes) after going to sleep, until the final awakening. The number of awakening is the frequency of awakening between sleep onset and final awakening. The total sleep time is counted by accounting for time before sleep onset and wake time after sleep onset. The sleep efficiency is measured by dividing the total sleep time with the total time in bed (time between going to bed to the final awakening), then times by 100. The feeling refreshed and sleep quality is measured by 5-point Likert scale, ranging from 0 (Tired) to 4 (Refreshed) and 0 (Poor) to 4 (Good), subsequently.

#### Perceived Stress Questionnaire (PSQ)

The Perceived Stress Questionnaire (PSQ) is used for the stress measure. PSQ was constructed as a measure for psychosomatic research that emphasizes on experiences that may causes psychosomatic symptoms. It is focused on subjective perception and emotional response. There are seven factors that are measured by PSQ: harassment, irritability, loss of joy, fatigue, worry, tension, and overload. PSQ have 30 items with 4-point Likert scale from 1  =  almost never to 4  =  usually[35]. PSQ is correlated with anxiety (STAI), depression (CES-D), and somatic complaints[35].

#### Subjective Well-Being (SWB)

The Subjective Well-Being measure is adopted from The Day Reconstruction Method[36]. The adopted measure only used three items from the package that asked about the participant's overall satisfaction with life as a whole, life at home, and life at work. SWB measures people's general happiness or satisfaction with their life[37]. SWB may indicate whether the treatment has contributed to the participants' overall quality of life. Healthcare has been recommended to adopt SWB measure to assess whether their treatment has contributed to the patient's overall quality of life[38]. Besides, SWB is related to positive coping. Thus, SWB may also show the efficacy of the treatment in improving the participants' coping mechanism and their overall quality of life.

Furthermore, psychological problems investigated are correlated with SWB. Anxiety, depression, and stress are problems that emphasize negative affect, which obviously negatively correlated with SWB[38]. Additionally, there is an evident negative relationship between chronic pain and SWB[39]. Insomnia is also correlated with SWB[40]. It is especially detrimental to a person's well-being when they have a recurrent insomnia, experiencing it more than once[41].

#### Client Satisfaction Questionnaire-8 (CSQ-8)

To measure each participant' satisfaction to the treatment they received, we used the Client Satisfaction Questionnaire-8 (CSQ-8)[42]. CSQ-8 is a brief and easily understood measure for client satisfaction. The measure has 8 items with 4-point Likert scale, with score range from 8 to 32. Measuring participants' satisfaction level is important because satisfaction level was known to be associated with symptoms reduction[43].

### Treatments

The treatments are several multicomponent group cognitive behavior therapies (GCBT) for the four specific psychological problems (anxiety, chronic pain, depression, and insomnia). This multicomponent GCBT is composed of techniques that have been shown to be effective from past research. There are several factors that we bear in mind when we composed the treatments, those are: evidence on the effectiveness of a technique, can be given in group format, easy to learn, and does not need much handwriting. These factors are important in the context of Indonesian elderly, whose education level is greatly varied and is not used to writing.

There are similarity and differences in the techniques used by the different treatment. The similarities are the use of psychoeducation on the specific problem, progressive muscular relaxation technique, cognitive techniques (ABCDE), and problem solving technique. Furthermore, all of the treatment also made use of some form of diary or monitoring technique. However, there are also differences that are related to the nature of the problem being treated. The treatment for anxiety aims to reduce the participants' overall anxiety level, thus its general application of the techniques. On the other hand, the treatment for chronic pain, depression, and insomnia is more specific. Chronic pain treatment involves teaching the participants to do their activities while in pain and to keep active. The depression treatment also pushes participants to be active with activity scheduling technique. In contrast, the insomnia treatment teaches participants to be less active with its sleep scheduling technique that promotes relaxation and regular sleeping habit.

The treatment is conducted in group format for every 3–4 days (twice a week), instead of the usual once a week meeting format. The twice a week meeting format was chosen to address the increasing likelihood of memory problems in the older adults population[5]. The group format is chosen for the development of an economical therapy. Each group consists of 4 to 7 participants. Participants who missed a session are given replacement session at the next session they attend to. The replacement session are conducted either individually or in group, depending on the number of participants that simultaneously missed the session. The treatment is conducted by five licensed clinical psychologists that received training in delivering CBT and is supervised by an experienced clinical psychologist with more than 10 years in professional private practice.

### Data analysis

The data analysis will use Paired Sample T-Test with bootstrap technique to compare the measures at pre-test and post-test. The bootstrapped statistical significance is used, instead of the usual normal curve statistical significance. The use of bootstrap has been recommended for experimental clinical psychology research, as the normal distribution assumption is rarely met[44]. Furthermore, bootstrap can also help improve the statistical analysis of small sample research (For example:[45], [46]). This research will use k = 5000 with 95% confidence intervals Bias-corrected and accelerated (BCa) [47]. BCa is argued to have a more accurate confidence interval rather than the percentile procedure[48].

## Results

The result will be discussed in two main sections, treatment attendance and treatment effect. The treatment attendance shows the rate of participation and how many participants missed the session. The treatment effect will be discussed by the main treatment outcome measures and several other measures that are unique to the treatment. Additionally, a measure of clients' satisfaction of the treatment will also be presented.

### Treatment attendance

Most of the participants attend all sessions without being late. The participants who did not attend any sessions are given materials they missed at the next session they attend to. For the anxiety treatment group, there is only one participant who missed a session. Then, there are three participants who missed sessions in the chronic pain treatment group; two participants missed one session and one participant failed to attend two sessions. Next, there are four participants that missed sessions in the depression treatment group. Two of them missed one session and the other two missed two sessions. Lastly, in the insomnia treatment group, there are two participants who failed to attend one session, and two participants who missed three sessions.

### Treatment effect

The treatment effect will be discussed on each psychological problem by a set of standard measures and additional secondary measures. Then, an overall picture of the treatments' efficacy will be shown. The result of the treatment effect will be discussed around changes that are statistically significant and effect size. Cohen's *d* is used to indicate effect size, with the following guideline: small (*d*<0.20), medium (*d* = 0.2–0.8), and large (*d*>0.80)[49].

### Multicomponent group cognitive behavior therapy (GCBT) for anxiety

The principal outcome measure for the multicomponent GCBT for anxiety treatment effect is the STAI-6. In addition, there is the PSWQ as an additional secondary outcome measure.

The table 1 shows the effect of the treatment. There is a statistically significant difference with large effect size between pre-test and post-test for STAI-6 (t(7) = 3.26, p<0.05, d = 1.48) and PSQ (t(7) = 3.62, p<0.01, d = 1.46). Chronic pain (CPAQ-8) shows almost no improvement due to the limited number of participant with chronic pain complaints. Whereas other measures shows modest improvement with medium effect size, ranging from *d* = 0.47 to 0.66.

**Table 1:** Treatment effect of the multicomponent GCBT for anxiety.

Measure	N	Pre-test	SD	Post-test	SD	Mean differences	BCa 95% Confidence Interval		t	Effect size (Cohen's *d*)
							Lower	Upper		
STAI-6	8	13.25	2.19	9.88	2.36	3.38	1.75	5.13	3.26*	1.48
CPAQ-8	3	36.00	7.00	36.33	1.15	−0.33	−4.67	4.00	−0.87	−0.07
CES-D 10	8	7.63	5.21	5.63	2.92	2.00	−2.13	6.23	1.02	0.47
ISI	8	3.25	1.98	2.25	2.25	1.00	−0.50	2.38	1.08	0.47
PSQ	8	0.33	0.07	0.23	0.07	0.10	0.05	0.14	3.62**	1.46
SWB	8	9.25	0.71	9.75	1.17	−0.50	−1.25	0.25	−0.94	−0.52
PSWQ	8	42.14	4.49	39.71	2.63	2.43	−0.71	5.29	1.38	0.66

* = p<0.05;

** = p<0.01, STAI-6: State-Trait Anxiety Inventory 6, CPAQ-8: Chronic Pain Acceptance Questionnaire 8, CES-D 10: Center for Epidemiologic Studies Depression Scale 10, ISI: Insomnia Severity Index, PSQ: Perceived Stress Questionnaire, SWB: Subjective Well-Being, PSWQ: Penn State Worry Questionnaire.

### Multicomponent group cognitive behavior therapy (GCBT) for chronic pain

The main outcome measure for the multicomponent GCBT for chronic pain is the CPAQ-8, with the set measures as secondary outcome measures. The table 2 shows the result of the treatment. There are two measures that reached statistical significance difference, CPAQ-8 (t(9) = −7.37, p<0.01, *d* = −3.16) and CES-D 10 (t(9) = 7.80, p<0.01, *d* = 1.98). The other measure also showed improvement with medium effect size, ranging from *d* = 0.22 to 0.67.

**Table 2:** Treatment effect of the multicomponent GCBT for chronic pain.

Measure	N	Pre-test	SD	Post-test	SD	Mean differences	BCa 95% Confidence Interval		t	Effect size (Cohen's *d*)
							Lower	Upper		
STAI-6	10	10.40	3.81	8.30	2.31	2.10	0.10	4.60	1.98	0.67
CPAQ-8	10	33.10	2.23	41.70	3.13	−8.60	−10.90	−6.60	−7.37**	−3.16
CES-D 10	10	6.00	2.21	1.90	1.91	0.09	0.01	0.18	7.80**	1.98
ISI	10	3.70	3.53	3.00	2.87	4.10	3.10	5.10	0.69	0.22
PSQ	10	0.28	0.17	0.20	0.06	0.70	−1.20	2.50	2.04	0.66
SWB	10	10.30	1.34	10.70	1.34	−0.40	−1.20	0.40	−0.77	−0.30

* = p<0.05;

** = p<0.01, STAI-6: State-Trait Anxiety Inventory 6, CPAQ-8: Chronic Pain Acceptance Questionnaire 8, CES-D 10: Center for Epidemiologic Studies Depression Scale 10, ISI: Insomnia Severity Index, PSQ: Perceived Stress Questionnaire, SWB: Subjective Well-Being.

### Multicomponent group cognitive behavior therapy (GCBT) for depression

The main outcome measure for the multicomponent GCBT for chronic pain is the CES-D 10 and BDI, with the set measures as secondary outcome measures. The table 3 shows the result of the treatment. Almost all of the measures reached statistical significant difference with large effect size. The largest effect size is shown by BDI measure (t(9) = 4.70, p<0.05, *d = *1.75), followed by STAI- 8 (t(9) = 2.20, p<0.01, *d = *1.43), ISI (t(9) = 4.59, p<0.01, *d = *1.41), CPAQ-8 (t(8) = −7.37, p<0.01, *d = *−3.16), and CES-D 10 (t(9) = 7.80, p<0.01, *d = *1.98). PSQ and SWB showed slighter improvement. PSQ have medium effect size (*d = *0.50), while SWB have small effect size (*d = *−0.08).

**Table 3:** Treatment effect of the multicomponent GCBT for depression.

Measure	N	Pre-test	SD	Post-test	SD	Mean differences	BCa 95% Confidence Interval		t	Effect size (Cohen’s *d*)
							Lower	Upper		
STAI-6	10	12.10	3.90	7.60	2.17	0.071	2.90	6.10	2.20**	1.43
CPAQ-8	9	32.67	4.87	39.89	5.97	−7.22	−9.78	−4.78	−4.98**	−1.33
CES-D 10	10	11.50	5.23	6.10	5.86	5.4	3.30	7.60	3.88**	0.97
ISI	10	6.20	3.19	2.70	1.49	4.5	2.40	4.60	4.59**	1.41
PSQ	10	0.34	0.14	0.27	0.14	3.5	0.02	0.12	5.22	0.50
SWB	10	8.90	1.60	9.00	0.94	−0.10	−0.80	0.70	−0.22	−0.08
BDI	10	17.50	7.96	5.70	5.21	11.8	8.20	15.60	4.70*	1.75

* = p<0.05;

** = p<0.01, STAI-6: State-Trait Anxiety Inventory 6, CPAQ-8: Chronic Pain Acceptance Questionnaire 8, CES-D 10: Center for Epidemiologic Studies Depression Scale 10, ISI: Insomnia Severity Index, PSQ: Perceived Stress Questionnaire, SWB: Subjective Well-Being, BDI: Beck Depression Inventory

### Multicomponent group cognitive behavior therapy (GCBT) for insomnia

The treatment effect for the multicomponent GCBT for insomnia is chiefly observed by changes in ISI scores. In addition, there are several indicators that are gathered from sleep diary that is unique to the insomnia treatment, which are: time before sleep onset, total wake time after sleep onset, number of awakening, total sleep time, sleep efficiency, feeling refreshed after sleep, and sleep quality.

The table 4 shows the effect of the treatment. The Paired Sample T-Test with bootstrap showed statistically significant differences at post-test for the primary outcome measure, the ISI (t(9) = 2.99, p<0.05, *d = *1.61). The other measures also showed general improvements, although it does not reach statistical significance. PSQ showed good improvement with large effect size (*d = *0.99). This is followed by STAI-6 (*d* = 0.68), CPAQ-8 (*d = *−0.45), and CES-D10 (*d = *0.44) that showed acceptable improvement with medium effect size. Furthermore, there is slight improvement of SWB with small effect size (*d = *−0.09). Additionally, the secondary outcome measures from the sleep diary indicators also showed general trend of improvements with medium to large effect size.

**Table 4:** Treatment effect of the multicomponent GCBT for insomnia.

Measure	N	Pre-test	SD	Post-test	SD	Mean differences	BCa 95% Confidence Interval of the difference		t	Effect size (Cohen’s *d*)
							Lower	Upper		
STAI-6	10	12.70	3.77	10.50	2.59	2.20	0.30	4.90	1.83	0.68
CPAQ-8	8	30.88	9.08	33.88	2.23	−6.25	−9.38	2.12	−1.04	−0.45
CES-D 10	10	7.20	3.55	5.60	3.78	1.60	0.20	3.00	1.81	0.44
ISI	10	13.60	5.17	6.40	3.63	7.20	2.80	12.50	2.99*	1.61
PSQ	10	0.35	0.14	0.23	0.10	0.13	0.05	0.23	2.89	0.99
SWB	10	9.40	1.17	9.50	0.97	−0.10	−0.60	0.50	−0.29	−0.09
Time before sleep onset (minutes)	7	30.93	26.27	16.69	9.64	14.24	1.68	31.64	1.51	0.72
Total wake time after sleep onset (minutes)	7	68.00	88.12	37.55	45.25	0.83	−1.55	3.41	0.66	0.43
Number of awakening	7	2.57	2.38	1.74	1.69	30.45	−13.27	88.01	0.95	0.40
Total sleep time (hours)	7	5.77	0.53	5.53	1.03	0.24	−0.56	0.99	0.50	0.29
Sleep efficiency (%)	7	70.90%	0.20	87.03%	0.11	−0.16	−0.31	−0.02	−2.11	−1.00
Refreshing feeling	7	2.71	0.74	3.38	0.57	−0.67	−1.42	0.13	−1.80	−1.01
Sleep quality	7	2.88	0.84	3.31	0.61	−0.43	−1.30	0.50	−0.97	−0.59

* = p<0.05;

** = p<0.01, STAI-6: State-Trait Anxiety Inventory 6, CPAQ-8: Chronic Pain Acceptance Questionnaire 8, CES-D 10: Center for Epidemiologic Studies Depression Scale 10, ISI: Insomnia Severity Index, PSQ: Perceived Stress Questionnaire, SWB: Subjective Well-Being.

Refreshing feeling and sleep efficiency shows the strongest improvement among sleep diary indicators. Refreshing feeling is improved from an average of 2.71 to 3.38 (*d = *1.01) and sleep efficiency is improved from the average of 70.90% to 87.03% (*d = *−1.00), both with large effect size. In addition, there is also an improvement of sleep quality from 2.88 to 3.31 (*d = *−0.59) with medium effect size. There is also a half reduction of time before sleep onset (from M = 30.93 minutes to M = 16.69 minutes, *d = *0.72) and total wake time after sleep onset (from M = 68.00 minutes to M = 37.55 minutes, *d = *0.43), both with medium effect size. The number of awakening is also reduced from the average of 2.57 to 1.74 (*d = *0.40) with medium effect size. Furthermore, the total sleep time is slightly reduced from the average of 5.77 hours to 5.53 hours (*d = *0.29).

### Efficacy of multicomponent group cognitive behavior therapy (GCBT)

The treatment efficacies of the four therapies are examined through the changes from its principal outcome measure and other additional measures. Table 5 summarizes the treatment effect for the therapies discussed above. Based on the principal outcome measure, all treatments achieve statistical significance difference at post-test with large effect size. Furthermore, the treatments also successfully improved a range of secondary outcomes with medium to large effect size. For example, the GCBT for chronic pain also successfully reduced the depression (CES-D 10) level of the participants.

**Table 5:** Summary of treatment efficacy.

Treatment	Statistical significance	Large effect size	Medium effect size
GCBT for anxiety			
Principal outcome	STAI-6	STAI-6	
Secondary outcome	PSQ	PSQ	CES-D 10, ISI, SWB, PSWQ*
GCBT for chronic pain			
Principal outcome	CPAQ-8	CPAQ-8	
Secondary outcome	CES-D10	CES-D10	STAI-6, ISI, SWB
GCBT for depression			
Principal outcome	CES-D10	CES-D10	
Secondary outcome	STAI-6, CPAQ-8, ISI, BDI*	STAI-6, CPAQ-8, ISI, BDI*	PSQ
GCBT for insomnia			
Principal outcome	ISI	ISI	
Secondary outcome		PSQ, Sleep Efficiency*, Refreshing Feeling*	STAI-6, CPAQ-8, CES-D 10, Time before sleep onset (minutes)*, Total wake time after sleep onset (minutes)*, Number of awakening*, Total sleep time (hours)*, Sleep quality*

* = secondary outcome measure to complement the principal measure, STAI-6: State-Trait Anxiety Inventory 6, CPAQ-8: Chronic Pain Acceptance Questionnaire 8, CES-D 10: Center for Epidemiologic Studies Depression Scale 10, ISI: Insomnia Severity Index, PSQ: Perceived Stress Questionnaire, SWB: Subjective Well-Being, PSWQ: Penn State Worry Questionnaire, BDI: Beck Depression Inventory.

### Client satisfaction

The Client Satisfaction Questionnaire (CSQ-8) indicates the overall satisfaction of the participants regarding the treatment. Table 6 shows the descriptive result of the CSQ-8. Overall, the participants have high level of satisfaction for all the treatments (the maximum score is 32.00). In addition, there is no statistically significance difference on client satisfaction between treatments (F (4, 45)  =  0.40, p = 0.81).

**Table 6:** Descriptive result of the Client Satisfaction Questionnaire (CSQ-8).

Treatment	N	Mean	SD
GCBT for anxiety	8	27.50	2.20
GCBT for chronic pain	10	28.90	2.18
GCBT for depression	10	28.70	2.75
GCBT for insomnia	10	28.00	2.75

## Discussion

The present study is the first to provide evidence of a short multicomponent GCBT to treat various common psychological disorders in Indonesian older adults. This study is imperative in providing short term evidence based psychological therapy for Indonesian older adults. The main result of this study shows that short multicomponent GCBT is effective in reducing symptoms with statistical significance changes and large effect size. The participants are also satisfied with the treatment, shown by CSQ-8 scores and treatment attendance. Furthermore, the treatments are not only effective in reducing its principal measure, but also other additional measures as well. However, the result must be interpreted with consideration of the limited sample size.

This study also explored the different pattern of symptom reduction among treatments. The pattern may be explained by the nature of the disorder. The anxiety treatment group is primarily effective in reducing anxiety and stress. Participants with anxiety cannot effectively handle their everyday problem, which is measured by the PSQ. As the treatment teaches them skills to reduce their anxiety, they can handle their anxiety better and be calmer in facing everyday problem. Subsequently, they perceived their everyday problem is less stressful. This explanation can also be used to explain the symptom reduction pattern by the depression treatment group, but with an additional note on comorbidity. The depression treatment group is effective in the symptom reduction of depression, as well as anxiety, chronic pain, and insomnia. This shows that depression is a disorder with widespread consequences. It has been previously established that depression is related with anxiety and insomnia ([50], [51]). Depression may also reduce the person's acceptance to chronic pain, where both pointed to inactive life. The link between depression and chronic pain acceptance is further shown by a successful reduction of depression symptoms in chronic pain acceptance treatment group. The multicomponent GCBT for chronic pain push the participants to be active despite of their chronic pain. The push to lead an active life that is present in depression and chronic pain treatment group may explain the link. Furthermore, the increasing acceptance of chronic pain has been documented to reduce depression[23]. On the other hand, the treatment group for insomnia is only successful in reducing the principal outcome measure. This is due to its highly specific nature that focussed on sleep quality improvement skills, unlike the previously discussed treatments that have a more general format.

Additionally, this research also employs SWB measure as an indicator of wellness. Changes in SWB scores may show whether the treatment has contributed to the participant's overall satisfaction with life. The result shows slight to modest SWB improvement across treatment. This is not to be expected as the principal measure showed good improvement and SWB has been found to be correlated with anxiety[38], chronic pain[39]), depression[38], and insomnia[40]. It seems that improvement on the latter would not necessary result in SWB improvement. This result may support the argument for a Wellness Based Intervention (WBT), where the main concern is to increase well-being instead of alleviating source of discomfort, to be adopted in mental health care[52].

On the other hand, the multicomponent GCBT has successfully addressed the Indonesian cultural context, indicated by the high satisfaction and good attendance. Beside quantitative data that shows high satisfaction, there are also qualitative data that shows strong approval of the treatment format. Several participants commented that they like this form of therapy much better than individual therapy. Therapy in group format allows them to meet with new people and get to know more people. They also often discussed the therapy materials after the sessions and how to do the homework. Additionally, some of the therapeutic group become friends and meet regularly after the end of the therapy. This continuation is expected as the habit of regular social gatherings (*arisan* or *silaturahmi*) is very natural to them.

However, there are several limitations of this study that are important to be mentioned. The most obvious limitation is the lack of power to detect significant differences. This study would benefit much from a larger sample. Next, the limited sample size also does not allow a covariance method for correcting pre-test values for the different experimental groups. Then, the participants' willingness to participate and get improved may become a confounding factor. Lastly, the pre- post-test quasi experimental method used in this study cannot provide any statement on comparison of other version of CBT. Further research should use larger sample size to investigate the efficacy of this short multicomponent GCBT. Additionally, another research is also needed to compare the effectiveness of short multicomponent GCBT to the standard individual CBT.

Despite the limitations, this finding adds to the accumulating body of evidence that showed the effectiveness of GCBT for various psychological disorders across cultures. Beside Indonesia, GCBT has also been shown to be effective in China[53], Japan[54], Malaysia[16], and Pakistan[6]. Therefore, based on the evidence shown, it can be concluded that GCBT may be an acceptable starting point of affordable psychological therapy for Indonesian older adults.

## References

[1] Badan Pusat Statistik (2010) Statistik penduduk lanjut usia Indonesia. Jakarta: Badan Pusat Statistik. pp. 36–37/186.

[2] Pomerantz AM (2011) Clinical psychology: Science, practice, and culture. Thousand Oaks, CA: Sage Publications, Inc.

[3] Beck JS (1995) Cognitive therapy: Basics & beyond. New York: Guilford.

[4] Butler AC, Beck JS (2000) Cognitive therapy outcomes: a review of meta-analyses. The Journal of Norwegian Psychological Association 37: 1–9.

[5] Laidlaw K, Thompson LW, Gallagher-Thompson D, Dick-Siskin L (2003) Cognitive behaviour therapy with older people. West Sussex: John Wiley & Sons.

[6] Naeem F, Waheed W, Gobbi M, Ayub M, Kingdon D (2010) Preliminary evaluation of culturally sensitive CBT for depression in Pakistan: findings from developing culturally-sensitive CBT Project (DCCP). Behavioural and cognitive psychotherapy 39: 165–173. 21092353 10.1017/S1352465810000822

[7] Weingarden H, Marques L, Fang A, LeBlanc N, Buhlmann U, et al (2011) Culturally Adapted Cognitive Behavioral Therapy for Body Dysmorphic Disorder: Case Examples. International Journal of Cognitive Therapy 4: 381–396. 25346783 10.1521/ijct.2011.4.4.381 PMC4205938

[8] Goodwin R, Giles S (2003) Social support provision and cultural values in Indonesia and Britain. Journal of Cross-Cultural Psychology 34 1–6.

[9] Suseno FM (1988) “Etika Jawa”: Sebuah Analisa Falsafi tentang Kebijaksanaan Hidup Jawa. Jakarta: Gramedia.

[10] Poedjiastutie D (2009) Culture shock experienced by foreign students studying at Indonesian Universities. TEFLIN Journal 20: 25–36.

[11] Knight BG, Kaskie B, Shurgot GR, Dave J (2006) Improving mental health of older adults In: Birren JE, Schaie KW, editors. Handbook of the psychology of aging. 6 ed. London: Elsevier Academic Press.

[12] Cohen CI, Magai C, Yaffee R, Walcott-Brown L (2006) The prevalence of anxiety and associated factors in a multiracial sample of older adults. Psychiatr Serv 57: 1719–1725. 17158485 10.1176/ps.2006.57.12.1719

[13] Won AB, Lapane K, Vallow S, Schein J, Morris JN, et al (2004) Persistent nonmalignant pain and analgesic prescribing patterns in elderly nursing home residents. J Am Geriatr Soc 52: 867–874. 15161448 10.1111/j.1532-5415.2004.52251.x

[14] Boswell EB, Stoudemire A (1996) Major depression in the primary care setting. Am J Med 101: 3S–9S. 10.1016/s0002-9343(96)00392-0 9012605

[15] Foley DJ, Monjan AA, Brown SL, Simonsick EM, Wallace RB, et al (1995) Sleep complaints among elderly persons: an epidemiologic study of three communities. Sleep: Journal of Sleep Research & Sleep Medicine 18: 425–432. 10.1093/sleep/18.6.425 7481413

[16] Mukhtar F, Oei TPS, Yacoob MJM (2011) Effectiveness of Group Cognitive Behaviour Therapy Augmentation in reducing negative cognitions in the treatment of depression in Malaysia ASEAN Journal of Psychiatry 12. 10.1016/j.ajp.2011.04.002 23051078

[17] Greenhoot AF (2003) Design and Analysis of Experimental and Quasi-Experimental Investigations. In: Roberts MC, Ilardi SS, editors. Handbook of Research Methods in Clinical Psychology. Oxford: Blackwell.

[18] Spielberger CD (1983) Manual for the state trait anxiety inventory (form Y) Palo Alto. CA: Mind Garden.

[19] Tluczek A, Henriques JB, Brown RL (2009) Support for the reliability and validity of a six-item State Anxiety Scale derived from the State-Trait Anxiety Inventory. J Nurs Meas 17: 19–28. 19902657 10.1891/1061-3749.17.1.19 PMC2776769

[20] Marteau TM, Bekker H (1992) The development of a six items short form of the state scale of the Spielberger State-Trait Anxiety Inventory (STAI). British Journal of Clinical Psychology 31: 301–306. 1393159 10.1111/j.2044-8260.1992.tb00997.x

[21] Meyer TJ, Miller ML, Metzger RL, Borkovec TD (1990) Development and validation of the Penn State Worry Questionnaire. Behaviour Research and Therapy 28: 487–495. 2076086 10.1016/0005-7967(90)90135-6

[22] Vowles KE, McCracken LM (2008) Acceptance and values-based action in chronic pain: a study of treatment effectiveness and process. Journal of Consulting and Clinical Psychology 76: 397–407. 18540733 10.1037/0022-006X.76.3.397

[23] McCracken LM, Vowles KE, Eccleston C (2004) Acceptance of chronic pain: Component analysis and a revised assessment method. Pain 107: 159–166. 14715402 10.1016/j.pain.2003.10.012

[24] Fish RA, McGuire B, Hogan M, Morrison TG, Stewart I (2010) Validation of the Chronic Pain Acceptance Questionnaire (CPAQ) in an internet sample and development and preliminary validation of the CPAQ-8. Pain 149: 435–443. 20188472 10.1016/j.pain.2009.12.016

[25] Radloff LS (1977) The CES-D scale: A self-report depression scale for research in the general population. Applied Psychological Measurement 1: 385–401.

[26] Shafer AB (2006) Meta-analysis of the factor structures of four depression questionnaires: Beck, CES-D, Hamilton, and Zung. Journal of Clinical Psychology 62: 123–146. 16287149 10.1002/jclp.20213

[27] Beekman ATF, Deeg DJH, Van Limbeek J, Braam AW, De Vries MZ, et al (1997) Criterion validity of the Center for Epidemiologic Studies Depression scale (CES-D): results from a community-based sample of older subjects in the Netherlands. Psychological Medicine 27: 231–235. 9122304 10.1017/s0033291796003510

[28] Irwin M, Artin KH, Oxman MN (1999) Screening for depression in the older adult: Criterion validity of the 10-Item Center for Epidemiological Studies Depression Scale (CES-D). Arch Intern Med 159: 1701–1704. 10448771 10.1001/archinte.159.15.1701

[29] Schulz R, Beach SR, Ives DG, Martire LM, Ariyo AA, et al (2000) Association between depression and mortality in older adults: The cardiovascular health study. Arch Intern Med 160: 1761–1768. 10871968 10.1001/archinte.160.12.1761

[30] Beck AT, Ward C, Mendelson M (1961) Beck Depression Inventory (BDI). Arch Gen Psychiatry 4: 561–571. 13688369 10.1001/archpsyc.1961.01710120031004

[31] Suwantara JR, Lubis DU, Rusli E (2005) Evaluasi Beck Depression Inventory sebagai sarana untuk mendeteksi depresi. Jurnal Psikologi Sosial 12: 69–77.

[32] Morin CM, Espie CA (2004) Insomnia: A clinical guide to assessment and treatment. New York: Kluwer Academic Publishers.

[33] Morin CM, Belleville G, Belanger L, Ivers H (2011) The Insomnia Severity Index: psychometric indicators to detect insomnia cases and evaluate treatment response. Sleep 34: 601–608. 21532953 10.1093/sleep/34.5.601 PMC3079939

[34] Edinger JD, Bonnet MH, Bootzin RR, Doghramji K, Dorsey CM, et al (2004) Derivation of research diagnostic criteria for insomnia: Report of an American Academy of Sleep Medicine Work Group. Sleep 27: 1567–1596. 15683149 10.1093/sleep/27.8.1567

[35] Levenstein S, Prantera C, Varvo V, Scribano ML, Berto E, et al (1993) Development of the perceived stress questionnaire: A new tool for psychosomatic research. Journal of Psychosomatic Research 37: 19–32. 10.1016/0022-3999(93)90120-5 8421257

[36] Kahneman D, Krueger AB, Schkade D, Schwarz N, Stone AA (2004) A survey method for characterizing daily life experience: The day reconstruction Method (DRM). Science 306: 1776–1780. 15576620 10.1126/science.1103572

[37] Diener E, Oishi S, Lucas RE (2003) Personality, culture, and subjective well-being: Emotional and cognitive evaluations of life. Annu Rev Psychol 54: 403–425. 12172000 10.1146/annurev.psych.54.101601.145056

[38] Diener E, Suh EM, Lucas RE, Smith HL (1999) Subjective well-being: Three decades of progress. Psychological Bulletin 125: 276–302.

[39] Lucas R, Clark A, Georgellis Y, Diener E (2004) Unemployment Alters the Set Point for Life Satisfaction. Psychological Science 15: 8–13. 14717825 10.1111/j.0963-7214.2004.01501002.x

[40] Hamilton NA, Gallagher MW, Preacher KJ, Stevens N, Nelson CA, et al (2007) Insomnia and well-being. J Consult Clin Psychol 75: 939–946. 18085910 10.1037/0022-006X.75.6.939

[41] Karlson CW, Gallagher MW, Olson CA, Hamilton NA (2012) Insomnia symptoms and well-being: Longitudinal follow-up. Health Psychol: in press. 10.1037/a0028186 22746259

[42] Larsen DL, Attkisson CC, Hargreaves WA, Nguyen TD (1979) Assessment of client/patient satisfaction: Development of a general scale. Evaluation and Program Planning 2: 197–207. 10245370 10.1016/0149-7189(79)90094-6

[43] Attkisson CC, Zwick R (1982) The client satisfaction questionnaire: Psychometric properties and correlations with service utilization and psychotherapy outcome. Evaluation and Program Planning 5: 233–237. 10259963 10.1016/0149-7189(82)90074-x

[44] Wright DB, London K, Field AP (2011) Using bootstrap estimation and the plug-in principle for clinical psychology data. Journal of Experimental Psychopathology 2: 252–270.

[45] Jeon ST, Hamid J, Maurer D, Lewis TL (2010) Developmental changes during childhood in single-letter acuity and its crowding by surrounding contours. Journal of Experimental Child Psychology 107: 423–437. 20633893 10.1016/j.jecp.2010.05.009

[46] Kley H, Tuschen-Caffier B, Heinrichs N (2011) Safety behaviors, self-focused attention and negative thinking in children with social anxiety disorder, socially anxious and non-anxious children. Journal of Behavior Therapy and Experimental Psychiatry 43: 548–555. 21831344 10.1016/j.jbtep.2011.07.008

[47] Carvalho JP, Hopko DR (2011) Behavioral theory of depression: Reinforcement as a mediating variable between avoidance and depression. Journal of Behavior Therapy and Experimental Psychiatry 42: 154–162. 21315876 10.1016/j.jbtep.2010.10.001

[48] Efron B (2003) Second thoughts on the bootstrap. Statistical Science 18: 135–140.

[49] Cohen J (1988) Statistical power analysis for the behavioral sciences Hillsdale. NJ: Erlbaum.

[50] Jansson-Fröjmark M, Lindblom K (2008) A bidirectional relationship between anxiety and depression, and insomnia? A prospective study in the general population. Journal of Psychosomatic Research 64: 443–449. 18374745 10.1016/j.jpsychores.2007.10.016

[51] Taylor DJ, Lichstein KL, Durrence HH, Reidel BW, Bush AJ (2005) Epidemiology of insomnia, depression, and anxiety. Sleep 28: 1457–1464. 16335332 10.1093/sleep/28.11.1457

[52] Slade M (2010) Mental illness and well-being: the central importance of positive psychology and recovery approaches. BMC Health Services Research 10. 10.1186/1472-6963-10-26 PMC2835700 20102609

[53] Wong DF (2008) Cognitive behavioral treatment groups for people with chronic depression in Hong Kong: a randomized wait-list control design. Depression and Anxiety 25: 142–148. 17340612 10.1002/da.20286

[54] Fujisawa D, Nakagawa A, Tajima M, Sado M, Kikuchi T, et al.. (2010) Cognitive behavioral therapy for depression among adults in Japanese clinical settings: a single-group study. BMC Research Notes 3. 10.1186/1756-0500-3-160 PMC2887906 20529252

