# Correction: Preliminary Study on the Effectiveness of Short Group Cognitive Behavioral Therapy (GCBT) on Indonesian Older Adults

**DOI:** 10.1371/annotation/f1eba6ed-88d1-46e5-90e7-04ed13ac5439

**Published:** 2013-12-09

**Authors:** Dharmayati Bambang Utoyo, Edo Sebastian Jaya, Retha Arjadi, Lathifah Hanum, Kresna Astri, Maha Decha Dwi Putri

The first author's name was spelled incorrectly. The correct name is: Dharmayati Bambang Utoyo. The correct abbreviation in the Contributions section is DBU. The correct citation is: Utoyo DB, Jaya ES, Arjadi R, Hanum L, Astri K, et al. (2013) Preliminary Study on the Effectiveness of Short Group Cognitive Behavioral Therapy (GCBT) on Indonesian Older Adults. PLoS ONE 8(2): e57198. doi:10.1371/journal.pone.0057198. There were additional errors in the Contributions section. The correct contributions are: Conceived and designed the experiments: DUL ESJ RA LH KA MDDP. Performed the experiments: ESJ RA LH KA MDDP. Analyzed the data: DUL ESJ RA. Contributed reagents/materials/analysis tools: DUL ESJ RA LH KA MDDP. Wrote the paper: DUL ESJ RA. 

